# Studies on the inhibitory effect of isavuconazole on flumatinib metabolism *in vitro* and *in vivo*

**DOI:** 10.3389/fphar.2023.1168852

**Published:** 2023-05-04

**Authors:** Ya-nan Liu, Xinhao Xu, Jingjing Nie, Yingying Hu, Xuegu Xu, Ren-ai Xu, Xiaoxiang Du

**Affiliations:** ^1^ Department of Pharmacy, The First Affiliated Hospital of Wenzhou Medical University, Wenzhou, Zhejiang, China; ^2^ School of Pharmaceutical Sciences, Wenzhou Medical University, Wenzhou, Zhejiang, China; ^3^ Department of Pharmacy, The Third Affiliated Hospital of Wenzhou Medical University, Wenzhou, Zhejiang, China; ^4^ Department of Pharmacy, The Eye Hospital of Wenzhou Medical University, Wenzhou, Zhejiang, China

**Keywords:** flumatinib, drug-drug interaction, isavuconazole, inhibition mechanism, UPLC-MS/MS

## Abstract

As the validated agent for the treatment of chronic myelogenous leukemia (CML), flumatinib is a novel oral tyrosine kinase inhibitor (TKI) with higher potency and selectivity for BCR-ABL1 kinase compared to imatinib. Many patients experience aspergillosis infection and they may start using isavuconazole, which is an inhibitor of CYP3A4. However, there is no study on their interaction *in vitro* and *in vivo*. In the present study, the concentrations of flumatinib and its major metabolite M1 were rapidly determined using an stable ultra-performance liquid chromatography tandem mass spectrometry (UPLC-MS/MS) method. The half-maximal inhibitory concentration (IC_50_) was 6.66 μM in human liver microsomes (HLM), while 0.62 μM in rat liver microsomes (RLM) and 2.90 μM in recombinant human CYP3A4 (rCYP3A4). Furthermore, the mechanisms of inhibition of flumatinib in human liver microsomes, rat liver microsomes and rCYP3A4 by isavuconazole were mixed. Moreover, ketoconazole, posaconazole, and isavuconazole showed more potent inhibitory effects than itraconazole, fluconazole, and voriconazole on HLM-mediated flumatinib metabolism. In pharmacokinetic experiments of rats, it was observed that isavuconazole could greatly change the pharmacokinetic parameters of flumatinib, including AUC_(0−t),_ AUC_(0−∞),_ C_max_ and CLz/F, but had no effect on the metabolism of M1. According to the results of *in vitro* and *in vivo* studies, the metabolism of flumatinib was inhibited by isavuconazole, suggesting that isavuconazole may raise the plasma concentration of flumatinib. Thus, it is important to take special care of the interactions between flumatinib and isavuconazole in clinical applications.

## 1 Introduction

Chronic myelogenous leukemia (CML), also known as chronic granulocytic leukemia, usually occurs in or after middle age which is a slowly progressive disease of the blood and bone marrow (Deininger et al., 2000). A reciprocal chromosome translocation (Zhao et al., 2014; Miceli and Kauffman, 2015), called the Philadelphia chromosome, leads to a constitutive activation of the BCR-ABL tyrosine kinase, resulting in CML (Nowell and Hungerford, 1960; Rowley, 1973; Goldman and Melo, 2003; Druker, 2008). For CML, current therapeutic strategies to prevent the proliferation of cancer cells and activate subsequent apoptosis are to use of tyrosine kinase inhibitors (TKIs), which inhibit BCR-ABL phosphorylation (Nowell and Hungerford, 1960; Kabarowski and Witte, 2000; O'Dwyer, 2002; Okuda et al., 2001). Nowadays, resistance to imatinib has caused investigators to develop other novel tyrosine kinase inhibitors (Zhao et al., 2014).

Flumatinib is a new oral TKI that is more selective and potent for BCR-ABL1 kinase compared to imatinib (Zhang et al., 2021). It was proven that flumatinib had much stronger autophosphorylation blocking activity of BCR-ABL1 kinase than imatinib (Luo et al., 2010). Considered as a first-line treatment option, flumatinib can lead to higher response rates, faster and deeper responses for patients with chronic phase CML, which means better survival outcomes and safer therapeutic interruptions in the future. In addition, patients taking flumatinib had fewer adverse events of rash, neutropenia, edema, pain in extremities, anemia and hypophosphatemia at 12-month follow-up (Zhang et al., 2021).

The important cytochrome P450 (CYP450) enzyme participating in the metabolism of flumatinib is CYP3A4. There are two main metabolites after treatment with CYP3A4, namely, M1 (N-desmethyl flumatinib) and M3 (amide hydrolysis product) (Yang et al., 2012). M1 has been shown to have a similar pharmacodynamic activity to the parent drug (Yang et al., 2012). Consequently, the inducers or inhibitors of CYP3A4 could affect the blood concentration of flumatinib, thus affecting the drug efficacy and increasing the incidence of adverse reactions, such as thrombocytopenia, leukopenia and other hematologic abnormalities, gastrointestinal abnormalities such as diarrhea, abdominal pain and vomiting, and an increase in the incidence of infection.

Various microbial-induced infections are the most common complications of leukemia and may have a serious impact on the survival rate of patients, where fungal secondary infections are one of the most devastating infections (Yano et al., 1996). In 2015, isavuconazole was approvable to be the first-line option against invasive aspergillosis and used as an initial treatment for trichinosis when treating with amphotericin B is inappropriate (Maertens et al., 2016; Marty et al., 2016). Compared to voriconazole, the compound has a lower rate of neurological and hepatic adverse events (Maertens et al., 2016; Wilson et al., 2016; Cornely et al., 2018; Denis et al., 2018).

Isavuconazole is a substrate and moderate inhibitor of CYP3A4 (Townsend et al., 2017), and flumatinib is mainly metabolized by CYP3A4. Therefore, drug-drug interactions (DDIs) may occur when they are used in combination. In this study, it was investigated to determine that the inhibitory effect of isavuconazole on the metabolism of flumatinib in rat liver microsomes (RLM), human liver microsomes (HLM), and in recombinant human CYP3A4 (rCYP3A4). Furthermore, Sprague–Dawley (SD) male rats were researched to study the interaction between flumatinib and isavuconazole *in vivo*. The findings are anticipated to provide basic data to promote the clinical application of flumatinib favorably.

## 2 Materials and methods

### 2.1 Chemicals and reagents

**FIGURE 1:**
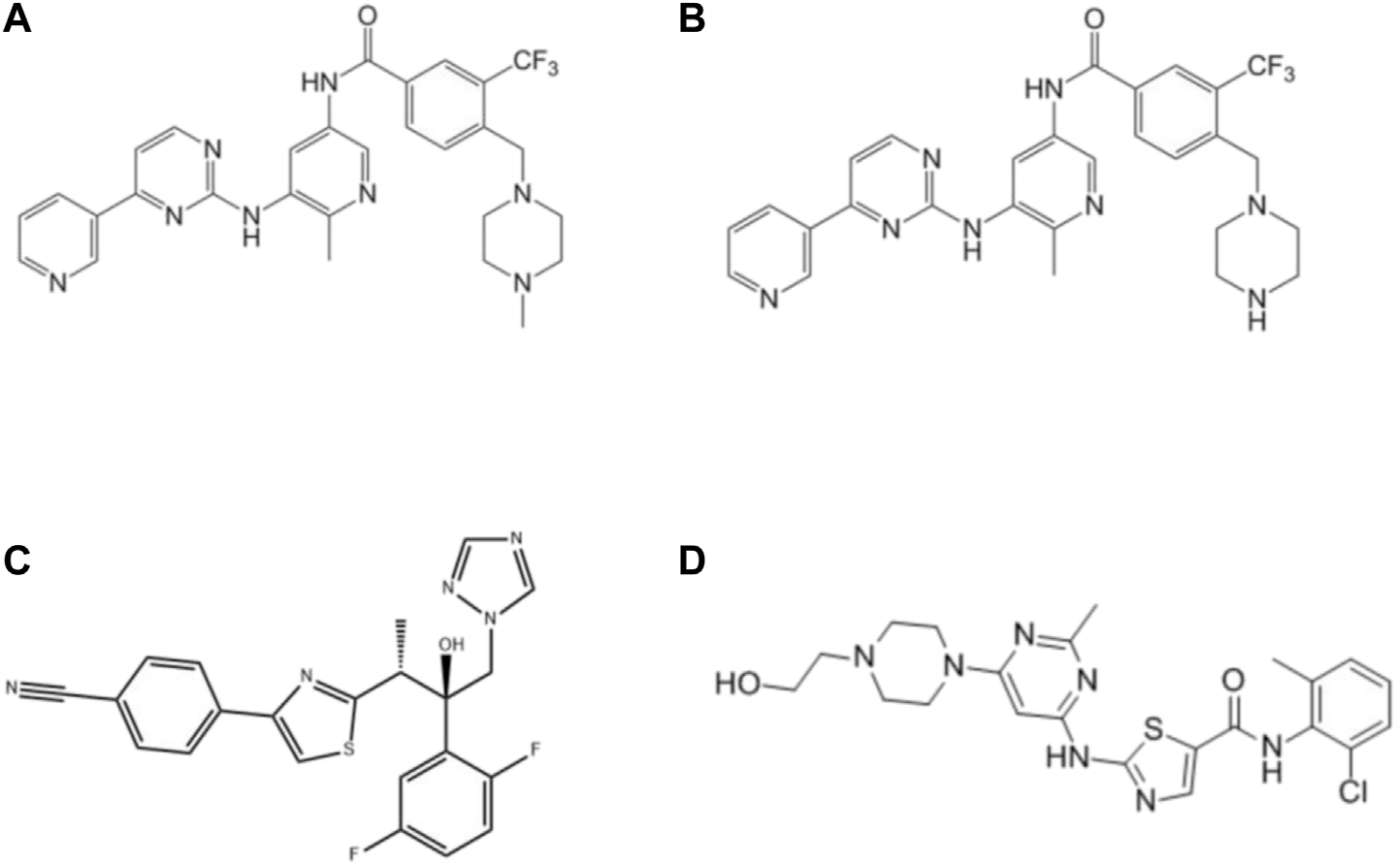
Structures of flumatinib **(A)**, M1 **(B)**, isavuconazole **(C)** and dasatinib (IS, **(D)** in this study.

Flumatinib (Figure 1A), and M1 (the main metabolite of flumatinib, Figure 1B) were provided kindly by Jiangsu Hansoh Pharmaceutical Group Co. Ltd. (Lianyungang, China). Isavuconazole (Figure 1C), dasatinib (used as internal standard, IS, Figure 1D) and other drugs were purchased from Shanghai Chuangsai Technology Co., Ltd. (Shanghai, China). HLM, RLM and rCYP3A4 were obtained from iPhase Pharmaceutical Services Co., Ltd. (Beijing, China). Reduced nicotinamide adenine dinucleotide phosphate (NADPH) was purchased from Roche Pharmaceutical Ltd. (Basel, Switzerland). All other unmentioned chemicals and solvents are available in analytical grade.

### 2.2 Equipment and operating conditions

Ultra-performance liquid chromatography tandem mass spectrometry (UPLC-MS/MS) equipped with a Waters Acquity UPLC BEH C18 column (2.1 mm × 50 mm, 1.7-μm particle size; Waters Corp., Millipore, Bedford, MA, United States) was used to detect the concentrations of flumatinib and M1. Quantification of the analytes was made by using a Waters XEVO TQS triple quadruple mass spectrometer with a multiple reaction monitoring (MRM) in positive ion mode. The mobile phase was consisted of 0.1% formic acid aqueous solution (A) and acetonitrile (B) at a flow rate of 0.4 mL/min. An elution program of 90% A (0–0.5 min), 90%–10% A (0.5–1.0 min), 10% A (1–1.5 min), 10%–90% A (1.5–1.6 min) and 90% A (1.6–2 min) was performed. The whole elution took 2 min. During the analysis, the temperature of the column and autosampler rack was controlled at 40°C and 10°C, respectively. In addition, optimized parameters for the mass spectra of flumatinib, M1 and dasatinib included: cone voltage were all 10 V, and collision energy was 20 eV, 25 eV, 20 eV, respectively. The monitoring transitions were *m/z* 563.1 → 463.0, *m/z* 549.3 → 462.9, and *m/z* 488.0 → 401.1 for flumatinib, M1 and dasatinib, respectively.

### 2.3 Determination of IC_50_ *in vitro*

An *in vitro* liver microsome incubation system was set up, then the drug was added to the system and incubated for 45 min as to determine the metabolism of the drug by the microsomes. With a total volume of 200 μL, the system contained 1 M PBS, flumatinib (1, 2, 5, 10, 20, 50 μM), HLM (0.3 mg/mL), and NADPH (1 mM) in the HLM, while the RLM system contained 1 M PBS, flumatinib (0.1, 0.5, 1, 2, 5, 10, 20 μM), RLM (0.4 mg/mL) and NADPH (1 mM). In addition, the rCYP3A4 system contained 1 M PBS, flumatinib (0.05, 0.1, 0.2, 0.5, 1, 2 μM), rCYP3A4 (0.5 pM) and NADPH (1 mM). The three systems were used to detect the Michaelis-Menten constant (K_m_) values of flumatinib in HLM, RLM and rCYP3A4, respectively. The NADPH-free mixture was preincubated at 37°C for 5 min, followed by the addition of 1 mM NADPH to activate the reaction. The mixture was incubated at 37 °C for 40 min until 20 μL of IS solution (200 ng/mL) and 400 μL of cool acetonitrile were added. It was then centrifuged at 13,000 rpm for 5 min. After that, 100 μL of the supernatant was taken to conduct the analysis by UPLC-MS/MS.

For the detection of possible DDIs between flumatinib and isavuconazole, the concentrations of flumatinib were 2.17 µM, 29.28 µM, and 3.06 µM in RLM, HLM and rCYP3A4, respectively, according to their K_m_ values. Then, we determined the half-maximum inhibitory concentration (IC_50_) of isavuconazole, and the concentration of isavuconazole was set to 0, 0.01, 0.1, 1, 10, 25, 50 and 100 μM in HLM, RLM and rCYP3A4. Ketoconazole, a strong inhibitor for CYP3A4, was used as a positive control. In addition, several additional azoles (such as voriconazole, posaconazole, itraconazole and fluconazole) in RLM and HLM were also evaluated for their IC_50_ on flumatinib metabolism.

### 2.4 Inhibitory mechanism of flumatinib by isavuconazole *in vitro*

The IC_50_ curves for flumatinib were obtained from the incubation system described above. *In vitro* inhibition mechanisms were investigated in microsomes. In HLM, the concentrations of isavuconazole and ketoconazole were set to 0, 2.66, 6.66, 13.31 μM and 0, 0.0049, 0.049, 0.098 μM, respectively, and the concentrations of flumatinib were established as 1.46, 5.86, 11.71 and 29.28 μM. In RLM, the concentrations of isavuconazole and ketoconazole were set to 0, 0.15, 0.62, and 1.23 μM and 0, 0.08, 0.16, 0.32 μM, respectively, and the levels of flumatinib were established as 0.55, 1.1, 2.2, and 4.4 μM. In rCYP3A4, the concentrations of isavuconazole were set to 0, 1.16, 2.90, and 5.80 μM, and the levels of flumatinib were established as 0.33, 1.32, 2.64, and 6.60 μM.

### 2.5 *In vivo* inhibition of flumatinib by isavuconazole

Sprague–Dawley male rats (SD male rats, 200 ± 20 g) were purchased from the Animal Experimental Center of The First Affiliated Hospital of Wenzhou Medical University (Zhejiang, China). Ten SD rats were then divided randomly and equally into two groups (*n* = 5): 60 mg/kg single oral dose of flumatinib (Group A); orally taken 20 mg/kg isavuconazole and 60 mg/kg flumatinib (Group B). The rats in both groups started fasting for 12 h ahead of the experiment, but were free to water. During the experimental day, group B was orally administered with isavuconazole dissolved in 0.5% carboxymethylcellulose sodium salt (CMC-Na) solution, while group A was orally administered with the same dose of CMC-Na solution. After administration of isavuconazole or CMC-Na for 30 min, all groups were given 60 mg/kg flumatinib. Tail vein blood samples were collected from rats in both groups at multiple time points (0.67, 1, 1.5, 2, 3, 4, 6, 8, 12, and 24 h). Subsequently, an additional 300 μL acetonitrile and 20 μL dasatinib (200 ng/mL) were added to 100 μL plasma. Then the mixture was vortexed to mix thoroughly for 2 min and centrifuged at 13,000 rpm for 10 min to obtain the final supernatant for UPLC-MS/MS analysis.

### 2.6 Statistical analysis

IC_50_ and enzyme kinetic parameters were obtained using GraphPad Prism 8.0 software (GraphPad software Inc., CA, United States). Non-compartmental analysis was performed by using Drugs and Statistics (DAS) software (version 3.0 software, Shanghai University of Traditional Chinese Medicine, China) to obtain pharmacokinetic profiles. Mean plasma concentration-time curves were generated using Origin 8.0, and the parameters were statistically investigated between groups using independent samples *t*-test by SPSS 24.0 software (SPSS Inc., Chicago, IL, United States). All data were expressed as mean ± SD and *p* < 0.05 was considered statistically significant.

## 3 Results

### 3.1 Development of ultra-performance liquid chromatography tandem mass spectrometry to determine flumatinib and M1

A successful method for the quantification of flumatinib and its metabolite M1 in plasma was developed. Flumatinib, M1 and dasatinib were all effectively separated without interfering with each other, with retention times of 1.13, 1.13 and 1.19 min, respectively. In the range of 1–500 ng/mL for flumatinib and 1–100 ng/mL for M1, the standard calibration curves for both flumatinib and M1 had correlation coefficients greater than 0.99. The lower limit of quantitation was 1 ng/mL for both compounds.

### 3.2 Effects of isavuconazole on the metabolism of flumatinib *in vitro*

**FIGURE 2:**
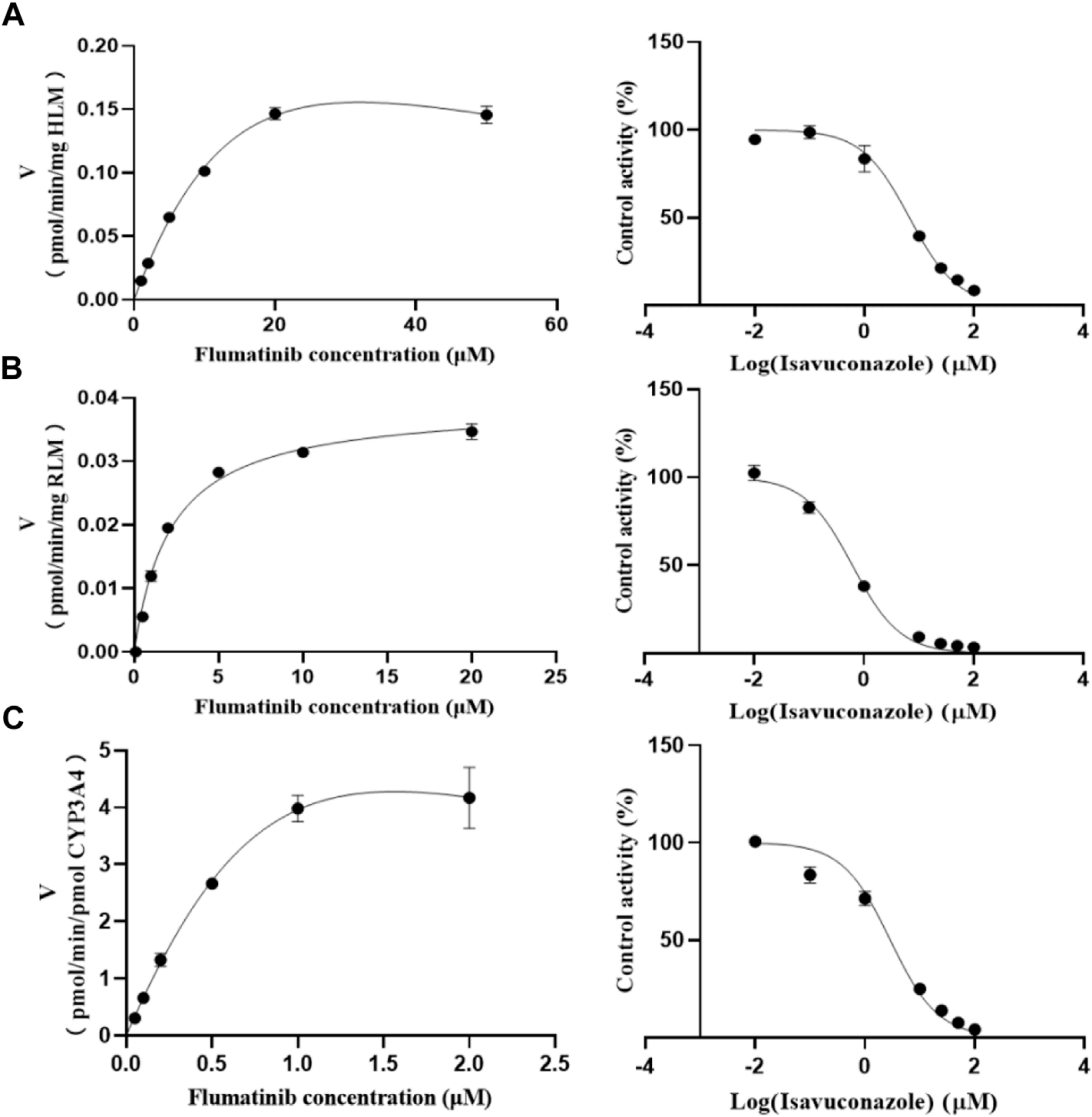
The Michaelis-Menten constant (K_m_) value and half-maximal inhibitory concentration (IC_50_) of flumatinib in HLM **(A)**, RLM **(B)** and rCYP3A4 **(C)**. Values are the mean ± SD, *N* = 3.

**FIGURE 3:**
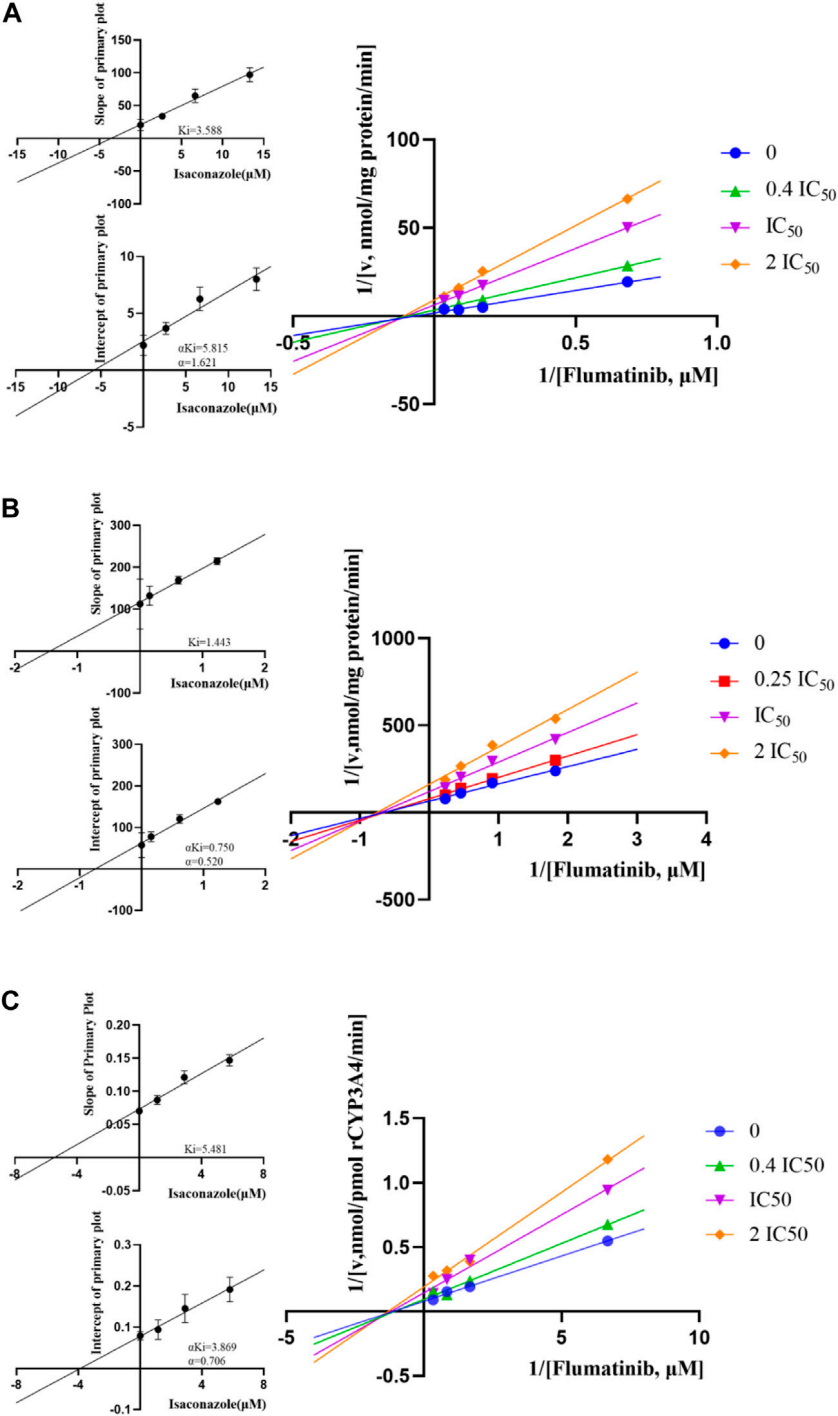
Lineweaver-Burk plot and the secondary plot for Ki in the inhibition of flumatinib metabolism by isavuconazole with various concentrations in HLM **(A)**, RLM **(B)** and rCYP3A4 **(C)**. Values are the mean ± SD, *N* = 3.

The K_m_ values of flumatinib in HLM, RLM and rCYP3A4 were 29.28 μM, 2.20 μM and 3.06 µM, respectively. The obtained IC_50_ by calculation were 6.66 μM, 0.62 μM, and 2.90 μM for HLM, RLM and rCYP3A4, respectively. These results could be found in Table 1 and Figure 2. The inhibitory mechanisms of isavuconazole on flumatinib in HLM, RLM and rCYP3A4 were further investigated, as shown in Figure 3. It was found that the inhibitory mechanisms of isavuconazole on flumatinib metabolism were mixed inhibitory mechanisms. The constant inhibition Ki of isavuconazole on flumatinib in HLM, RLM and rCYP3A4, were 3.59, 1.44, and 5.48 μM, respectively. The Ki value of isavuconazole in RLM was significantly lower than that in HLM and rCYP3A4. The lower Ki suggested that isavuconazole in RLM had stronger binding affinity with flumatinib than that in HLM and rCYP3A4.

**TABLE 1:** The IC_50_ values and inhibitory effect of isavuconazole on flumatinib metabolism in HLM RLM, and rCYP3A4.

	IC_50_ values (μM)	K_m_ (μM)	Inhibition type	Ki (μM)	αKi (μM)	α
HLM	6.66	29.28	Mixed inhibition	3.59	5.81	1.62
RLM	0.62	2.20	Mixed inhibition	1.44	0.75	0.52
rCYP3A4	2.90	3.06	Mixed inhibition	5.48	3.87	0.71

In addition, the IC_50_ values of ketoconazole in HLM and RLM were 0.05 μM and 0.16 µM, respectively, with the Ki value of 0.53 μM and 0.33 μM. And, the inhibitory mechanisms of ketoconazole on flumatinib metabolism were mixed inhibitory mechanisms in both HLM and RLM. These results were presented in Supplementary Table S1 and Supplementary Figure S1. As for the IC_50_ values of other azoles (voriconazole, posaconazole, itraconazole and fluconazole) in RLM and HLM, could be found in Supplementary Table S2 and Supplementary Figure S2.

### 3.3 Isavuconazole significantly alters the major pharmacokinetic profiles of flumatinib in SD rats

**FIGURE 4:**
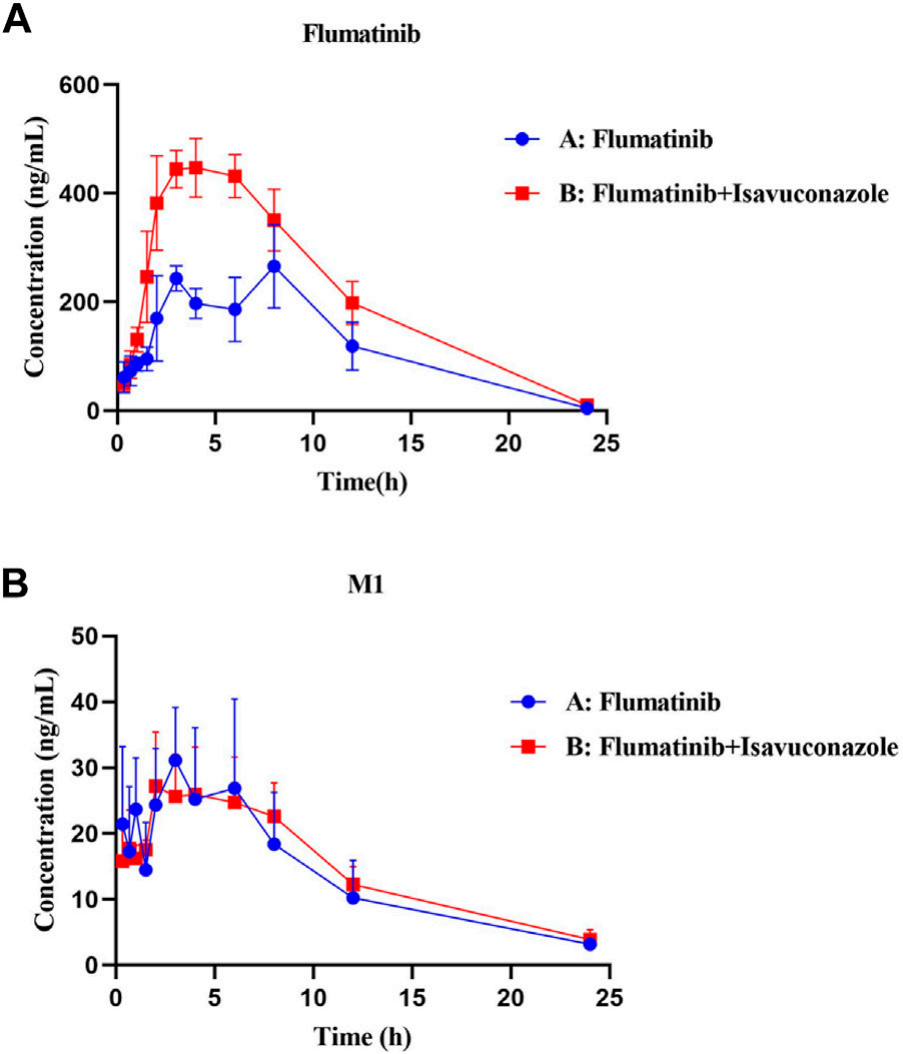
Mean plasma concentration-time curves of flumatinib **(A)** and M1 **(B)** in two rat groups (group A: 60 mg/kg flumatinib dosed orally; group B: 20 mg/kg isaconazole and 60 mg/kg flumatinib dosed orally) (*n* = 5, Mean ± SD).

The pharmacokinetic parameters of flumatinib and M1 were shown in Tables 2, 3, with their mean plasma concentration-time curves *in vivo* were presented in Figure 4. When isavuconazole and flumatinib were both administered orally, the values of AUC_(0–t)_, AUC_(0–∞)_ and C_max_ were 1.72, 1.88 and 1.70-fold, while CLz/F was 48% lower than those of group A, respectively (all *p* < 0.05). However, it was not found the pharmacokinetic parameters of M1 were significantly different between the two groups of rats.

**TABLE 2:** The main pharmacokinetic parameters of flumatinib in SD rats in two rat groups (group A: 60 mg/kg flumatinib dosed orally; group B: 20 mg/kg isavuconazole and 60 mg/kg flumatinib dosed orally) (*n* = 5, Mean ± SD).

Parameters	Flumatinib	Isavuconazole + flumatinib
AUC_0→t_ (ng/mL•h)	2747.05 ± 887.41	4728.31 ± 1290.75 *
AUC_0→∞_ (ng/mL•h)	3044.19 ± 663.97	5714.58 ± 744.11 ***
MRT_0→t_ (h)	6.82 ± 1.94	6.87 ± 1.33
MRT_0→∞_ (h)	7.66 ± 2.01	9.20 ± 3.59
t_1/2_ (h)	4.04 ± 2.65	4.88 ± 2.91
T_max_ (h)	6.00 ± 2.74	4.40 ± 1.52
CLz/F (L/h/kg)	20.42 ± 4.14	10.63 ± 1.27**
C_max_ (ng/mL)	281.98 ± 36.77	480.96 ± 33.31 ***

Compared with the Group A, *p < 0.05; **p < 0.01; ***p < 0.001. AUC, area under the plasma concentration-time curve; MRT, mean residence time: t_1/2_, elimination half time; T_max_, peak time; CLz/F, plasma clearance; C_max_, maximum plasma concentration.

**TABLE 3:** The main pharmacokinetic parameters of M1 in SD rats in two rat groups (group A: 60 mg/kg flumatinib dosed orally; group B: 20 mg/kg isavuconazole and 60 mg/kg flumatinib dosed orally) (*n* = 5, Mean ± SD).

Parameters	Flumatinib	Isavuconazole + flumatinib
AUC_0→t_ (ng/mL•h)	281.73 ± 134.98	351.54 ± 49.80
AUC_0→∞_ (ng/mL•h)	313.56 ± 133.64	395.21 ± 63.10
MRT_0→t_ (h)	6.73 ± 1.68	8.20 ± 0.19
MRT_0→∞_ (h)	8.86 ± 1.39	11.15 ± 1.53
t_1/2_ (h)	5.28 ± 1.24	7.29 ± 2.01
T_max_ (h)	2.60 ± 1.14	5.00 ± 2.45
CLz/F (L/h/kg)	236.22 ± 140.35	154.82 ± 23.55
C_max_ (ng/mL)	19.86 ± 32.81	31.88 ± 3.98

AUC, area under the plasma concentration-time curve; MRT, mean residence time: t_1/2_, elimination half time; T_max_, peak time; CLz/F, plasma clearance; C_max_, maximum plasma concentration.

## 4 Discussion

CML is a myeloproliferative neoplasm that constitutes roughly 15% of newly diagnosed leukemia cases in adults (Jabbour and Kantarjian, 2020). As a novel oral BCR-ABL1 TKI, flumatinib has shown superior efficacy in treating chronic-phase CML compared to imatinib (Zhao et al., 2014). Nevertheless, since CYP3A4 is the main metabolizing enzyme of flumatinib, interactions between flumatinib and other drugs must be taken into account carefully.

Isavuconazole, an azole antifungal drug, is suitable for the first-line option to treat invasive aspergillosis and the second-line choice for treating mucormycosis (Ellsworth and Ostrosky-Zeichner, 2020). Like other azoles, isavuconazole inhibits cytochrome P450 (CYP)–dependent 14α-lanosterol demethylation, which is essential for fungal cell membrane ergosterol synthesis. It is metabolized by the liver through CYP3A4 and CYP3A5 and UDP-glucuronosyltransferase (UGT) (Ellsworth and Ostrosky-Zeichner, 2020). The interaction between isavuconazole and a wide range of drugs must be considered since it is a moderate CYP3A4 inhibitor (Townsend et al., 2017). The plasma levels of cyclosporine A, tacrolimus, sirolimus, and mycophenolate mofetil could be raised, when isavuconazole is co-administered (Miceli and Kauffman, 2015). According to the reports, when combined with isavuconazole, the AUC was increased by 29% for cyclosporine A, 125% for tacrolimus, and 84% for sirolimus (McCarthy et al., 2018). A decrease in plasma levels of digoxin, lopinavir and ritonavir by isavuconazole has also been reported (McCarthy et al., 2018). In addition, isavuconazole has been recently shown to be the weakest inhibitor of CYP3A4 substrate midazolam among various azoles such as ketoconazole, voriconazole, fluconazole, itraconazole and posaconazole in clinical DDI study in healthy volunteers (Rohr and Mikus, 2020). Thus, monitoring of serum levels and adverse effects is necessary if these medications are administered with isavuconazole.

Although increased systemic exposure to flumatinib has been reported with voriconazole, the co-administration of flumatinib with isavuconazole has never been investigated (Chen et al., 2020). In the present study, an inhibition of flumatinib metabolism by isavuconazole was investigated *in vitro* and *in vivo*. Compared to the control group, it was demonstrated that isavuconazole inhibited flumatinib to 7.75% in RLM. *In vitro*, the IC_50_ value of flumatinib by isavuconazole in HLM, RLM and rCYP3A4 were 6.66 μM, 0.62 μM and 2.90 μM, respectively. It could be presumed that the metabolism of flumatinib *in vitro* was inhibited by isavuconazole. As a further study, the mechanisms of inhibition between these two drugs were investigated, which was revealed that the inhibition of flumatinib metabolism in three systems by isavuconazole was a mixed type. In addition, the constant inhibition Ki of isavuconazole on flumatinib in HLM, RLM, and rCYP3A4, were 3.59, 1.44, and 5.48 μM, respectively. The higher inhibition potential of isavuconazole in RLM with a lower Ki value pointed out that it is more likely to display DDIs in RLM than in HLM and rCYP3A4.

In addition, the inhibitory effects of five additional azoles on flumatinib metabolism were investigated in HLM and RLM, in which ketoconazole was used as a positive control for it was found to be the most potent inhibitor of flumatinib metabolism in RLM. The results clearly showed that *in vitro* inhibitory effects were found at distinct degrees. Of note, ketoconazole showed the more potent inhibitory effects than isavuconazole on flumatinib metabolism. Also, based on the IC_50_ values of HLM-mediated metabolism by all azoles, ketoconazole, posaconazole, and isavuconazole, had stronger inhibitory capacities than itraconazole, fluconazole, and voriconazole on the metabolism of flumatinib.

The AUC_(0−t)_, AUC_(0→∞)_ and C_max_ of flumatinib were increased by isavuconazole *in vivo*, and these parameters were 1.72, 1.88 and 1.70 times higher than those in the control group. Other pharmacokinetic important parameter, such as CLz/F, was decreased significantly in the combined dose group. No statistical significances were found between the combination and control groups in investigating the pharmacokinetic parameters of the metabolite M1. With these outcomes, it was clear that isavuconazole increased the exposure of flumatinib in rats.

Clinically, many patients may use flumatinib and triazole antifungal agents concurrently because secondary fungal infections are prone to occur during the use of flumatinib. Hence, there are profound clinical importance in studying their interactions. The results of the current study could also provide an accurate application basis for the combination of the two drugs in the future. Further research should be illustrated the impact of isavuconazole on the flumatinib in humans.

## 5 Conclusion

To summarize, isavuconazole had an inhibitory effect on flumatinib metabolism *in vivo* and *in vitro*. And, mechanisms of *in vitro* inhibition in HLM, RLM and rCYP3A4 were a mixture of competitive and non-competitive inhibition. In HLM-mediated metabolism by all azoles, ketoconazole, posaconazole, and isavuconazole, had stronger inhibitory capacities than itraconazole, fluconazole and voriconazole on the metabolism of flumatinib. In addition, it was demonstrated that isavuconazole remarkably changed the main pharmacokinetic profiles of flumatinib, increasing the systemic exposure in rats. The current study found that the interaction between isavuconazole and flumatinib, which may be helpful for therapeutic drug monitoring, clinical dose reference and provide a valuable tool for drug-drug interactions. The rat *in vivo* study does not predict accurately the *in vivo* situation in humans, since the contribution of rat CYP3A enzymes to metabolism of CYP3A4 substrates is different from that in humans. Further research should be done to confirm this result in clinical studies.

## Data Availability

The raw data supporting the conclusion of this article will be made available by the authors, without undue reservation.
