# Supplementary material for: Studies on the inhibitory effect of isavuconazole on flumatinib metabolism in vitro and in vivo
Source: Front Pharmacol. 2023 May 4;14:1168852. doi: 10.3389/fphar.2023.1168852 (PMC10192561; doi:10.3389/fphar.2023.1168852)
Supplement: Supplementary file 1 [file DataSheet1.pdf]

Supplementary information

Studies on the inhibitory effect of isavuconazole on flumatinib metabolism *in vitro* and *in vivo*

Supplementary figures: 2
Supplementary Tables: 2

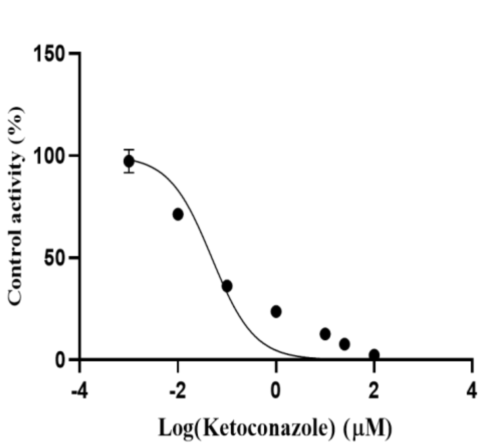

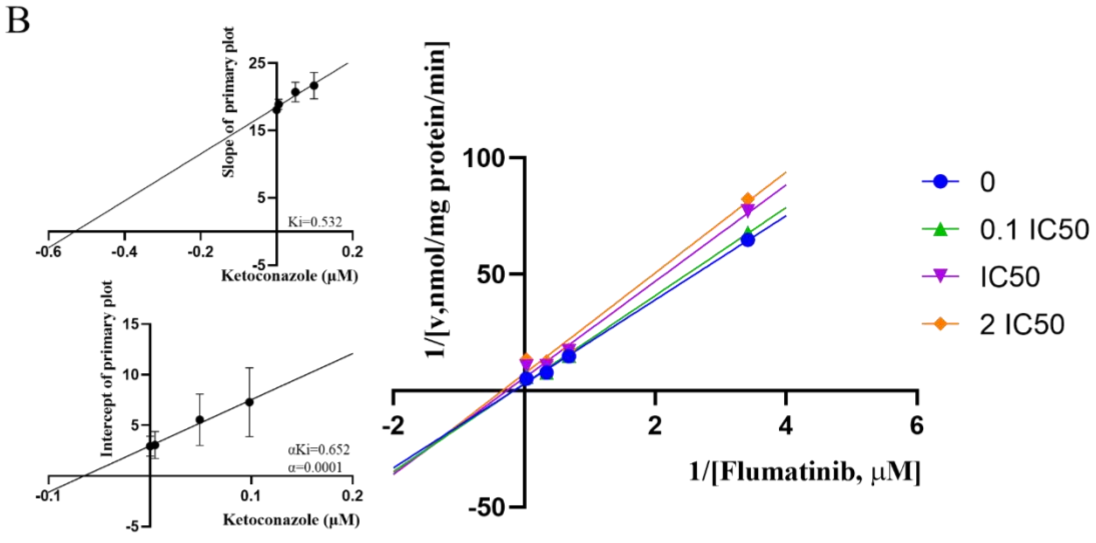

C

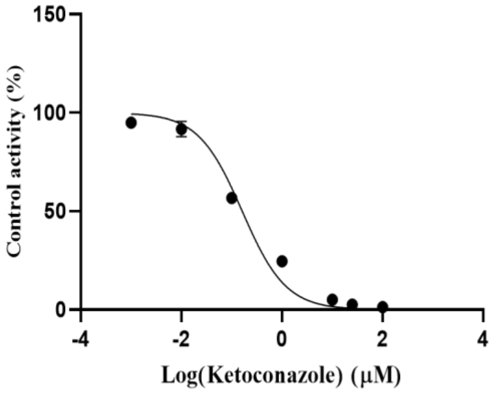

D

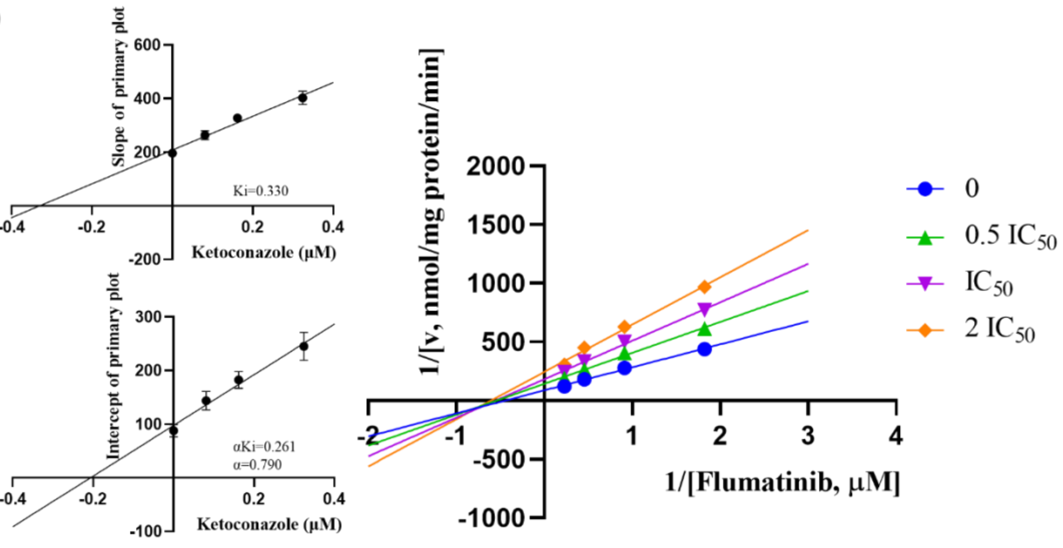

Supplementary Fig. S1. IC_{50}, lineweaver-Burk plot and the secondary plot for Ki in the inhibition of flumatinib metabolism by ketoconazole with various concentrations in HLM (A and B) and RLM (C and D). Values are the mean ± SD, N = 3.

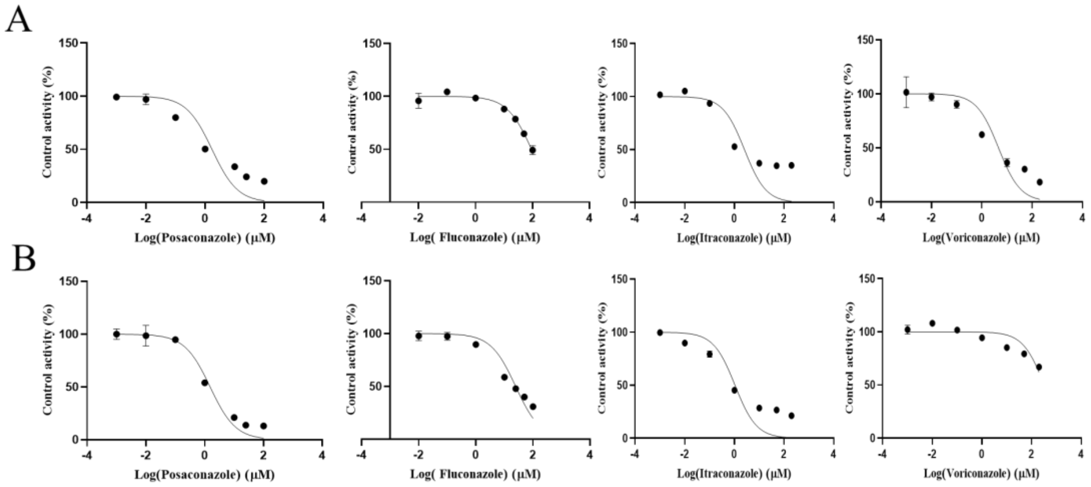

Supplementary Fig. S2. IC_{50} of posaconazole, fluconazole, itraconazole and voriconazole on flumatinib in HLM (A) and RLM (B). Values are the mean ± SD, N=3.

Supplementary Table S1. The IC_{50} values and inhibitory effect of ketoconazole on flumatinib metabolism in HLM and RLM.

	IC_{50} values (μM)	Inhibition type	Ki (μM)	αKi (μM)	α
HLM	0.05	Mixed inhibition	0.53	0.65	0.00
RLM	0.16	Mixed inhibition	0.33	0.26	0.79

Supplementary Table S2. The IC_{50} values of posaconazole, fluconazole, itraconazole, and voriconazole on flumatinib metabolism in HLM and RLM.

Drug	HLM-IC_{50}	RLM-IC_{50}
posaconazole	1.715	1.529
fluconazole	92.63	24.79
itraconazole	11.55	1.058
voriconazole	324.6	12.41
